# Identification of sensitive indicators in immune response for leprosy affected patients: An observational clinical study of safety and immunogenicity of influenza vaccine

**DOI:** 10.1097/MD.0000000000026744

**Published:** 2021-08-06

**Authors:** Yi Zheng, Hong-yi Xing, Zheng-Gang Zhu, Hong-Hao Zhu, Fang Zhang, Xia Gao, Jun Gao, Quan Hu, Yuan Fang

**Affiliations:** a Department of Leprosy, Wuhan Institute of Dermatology and Venereology, Wuhan, China; b Department of Neurology, Union Hospital, Tongji Medical College, Huazhong University of Science and Technology, Wuhan, China; c Department of Immunization, Wuhan Centers for Disease Prevention and Control, Wuhan, China.

**Keywords:** immunogenicity, influenza vaccines, interleukin-17, interleukin-6, leprosy, safety, vaccine

## Abstract

Cured leprosy patients have special physical conditions, which could pose challenges for safety and immunogenicity after immunization. We performed an observational clinical study aimed to identify the safety and immunogenicity of influenza vaccine in cured leprosy patients. A total of 65 participants from a leprosarium were recruited into leprosy cured group or control group, and received a 0.5 ml dose of the inactivated split-virion trivalent influenza vaccine and a follow-up 28 days proactive observation of any adverse events. Hemagglutination and hemagglutination inhibition test was performed to evaluate serum antibody titer, flow cytometry was conducted to screen of cytokines level. The total rate of reactogenicity was 0.0% [0/41] in leprosy cured group and 37.5% [9/24] in control group. The seroconversion rate for H1N1 was difference between leprosy cured group and control group (41.83% vs 79.17%, *P* = .0082), but not for H3N2 (34.25% vs 50.00%, *P* = .4468). At day 0, leprosy cured group have relatively high concentration of interleukin-6, interleukin-10, tumor necrosis factor, interferon-γ, and interleukin-17 compared to control group. The interleukin-2 concentration increased 2 weeks after vaccination compared to pre-vaccination in leprosy cured group, but declined in control group (0.92 pg/ml vs −0.02 pg/ml, *P* = .0147). Leprosy cured group showed a more rapid down-regulation of interleukin-6 when influenza virus was challenged compared to control group (−144.38 pg/ml vs −11.52 pg/ml, *P* < .0001). Subgroup analysis revealed that the immunization administration declined interleukin-17 concentration in Tuberculoid type subgroup, but not in Lepromatous type subgroup or control group. Clinically cured leprosy patients are relatively safe for influenza vaccine. Leprosy cured patient have immune deficit in producing antibody. Interleukin-6 and interleukin-17 were 2 sensitive indicators in immune response for leprosy affected patients. The identification of indicators might be help management of leprosy and used as predictive markers in leprosy early symptom monitoring.

## 1 Introduction

Leprosy is an ancient infectious disease caused by *Mycobacterium leprae* and *Mycobacterium lepromatosis*. Since late 20th century, leprosy patient could be cured by multi-drug therapy (MDT), but leprosy is still unclear on various aspects including transmission, immunology, nerve damage etc.^[1–3]^ Transmission is believed to occur through close contact with an infected person, but almost 95% of adults have native immunity to leprosy.^[4]^ Although leprosy is largely treated with MDT onset, many cured patients still suffer from life-long disability associated with the disease, and have to live in communities which composed largely of people affected by leprosy, also called leprosariums.^[5]^ Clinical manifestations of leprosy have been sufficiently studied for the past decades and there's increasing study and focus on gene and immunity related to leprosy in recent years. Leprosy is recognized as the first disease to be classified according to the host immune response. Thus, patients with Tuberculoid leprosy (TL) are characterized by a relevant T cell immune response, including interleukin-2 (IL-2), interleukin-4 (IL-4), interleukin-6 (IL-6), interleukin-10 (IL-10), tumor necrosis factor (TNF), interferon-γ (IFN-γ), and interleukin-17 (IL-17) and lymphotoxin, manifested by few cutaneous or neural lesions with little or no bacilli. In contrast, patients with Lepromatous leprosy (LL) present a greater humoral immune response, characterised by multiple lesions, high bacterial load, diminished or absent lymphocyte proliferation.^[6,7]^ The innate immune response to *M. leprae* infection involves both TLR1, TLR2 and NOD-like receptors.^[8,9]^ Various cytokines such as IFN-γ, IL-10 generated by the innate immune response have important roles against infection.^[10]^ The up-regulate production of various cytokine persist in leprosy patient even if *M. leprae* is controlled by MDT.^[11,12]^*M. leprae* as an immunity inducer, provide non-specific cross-defence protection against other unrelated pathogens.^[13]^

Inactivated influenza virus vaccine is recommended as an important influenza prevention strategy because of its safety and tolerability. Influenza virus vaccination elicits a measurable inflammatory response among health people, as increases in cytokines such as IL-6 and TNF-α within 48 hours.^[14,15]^ But many survivors from hundreds of leprosarium in China are disqualified from vaccination due to the unclear immunogenicity and safety of vaccination. Influenza vaccine usually provides short-term and strain specific humoral immunity, thus pose challenges for efficacy and safety for clinically cured leprosy patient. In the present study we conducted a clinical study to (1) Identify the safety and immunogenicity of influenza vaccine in cured leprosy patients; (2) Whether the immune deficiency or immunologic derangement of cured leprosy patient could affect host immune response against vaccination? (3) Further understanding of host defense of cured leprosy patients may provide new insights for the prevention, diagnosis and treatment of leprosy, or any other immune disease. Most of all, we hope to provides evidence to support that persons affected by leprosy should be treated equally in medical care, thus reduce stigma and discrimination and promote social inclusion.

## 2 Materials and methods

### 2.1 Patients and study design

The observational clinical study was conducted between November 15, 2017 and December 15, 2017. Participants including leprosy cured patient (leprosy cured group) and staff (control group) were recruited from Wuhan leprosarium. The leprosy cured standard consistent with “Clinical cure standard of leprosy” issued by Ministry of Public Health of China.^[16]^ All leprosy cured patients (68) and staff (33) from Wuhan leprosarium were initially classified as eligible, provided consent to participate. Subgroup analysis divided leprosy cured patient into 2 groups by leprosy type or whether suffering ulcer. TL and borderline TL were considered as Tuberculoid type (TT), and LL, borderline LL and midborderline leprosy were considered as Lepromatous type (LT). Participants were excluded if they had fever, or severe allergic history for vaccination, or thrombocytopenia or other disturbance of blood coagulation which would lead to muscle injection taboo, or serious cardiovascular disease, or receipt of vaccines within 2 weeks, or receipt of aspirin because of chronic diseases, or receipt cortisol (betamethasone, betamethasone, cortisone acetate, etc), or any other conditions that clinicians thought that they should be excluded. 5 ml whole blood is collected before vaccination and 14 days after vaccination, and stored at −70°C. The protocol of this study was approved by the Medical Research Review Board of researcher's affiliation conform to STROBE guidelines (WHCDCIRB-K-2017004), and written informed consent was obtained from all participants, and all participants were adults.

### 2.2 Vaccine

Subjects received intramuscular injections (deltoid muscle) of a single 0.5 ml dose of the inactivated split-virion trivalent influenza vaccine (“Vaxigrip”, Sanofi Pasteur) by trained nurses. The vaccine was provided in prefilled syringes of 0.5 ml (containing 15 mg hemagglutinin per strain) of A/Michigan/45/2015(H1N1)pdm09, A/HongKong/4801/2014(H3N2), and B/Brisbane/60/2008 in compliance with World Health Organization recommendations.^[17]^

### 2.3 Safety monitoring

A more proactive safety monitoring strategy was conducted. Before vaccination, a face -to-face survey was performed by clinician to collect the demographic and clinical information. Side effects were observed by clinician for 30 minutes after vaccination for both groups, and any adverse reactions were recorded by clinician during follow-up at 24 hours, 48 hours, 72 hours, 14 days and 28 days post-immunization. Safety information were collected including the occurrence, nature, duration, intensity, action taken, and relationship to vaccination of any solicited adverse event (AE), unsolicited AE, or serious adverse events (SAE). AEs and SAEs were recorded according to National Institute of Allergy and Infectious Diseases (NIAID)^[18]^ and “preventive vaccine clinical trials, adverse events grading guidelines” issued by the China Food and Drug Administration (CFDA).^[19]^ All SAEs were reported to the Ethical Review Committee and the drug adverse reaction monitoring system. The investigator categorized all AEs and SAEs as probably related, possibly related or not related to vaccine, according to the WHO standard.

### 2.4 Immunogenicity analysis

Immunogenicity assessments included: number and percentage of subjects with a serum hemagglutination-inhibiting (HAI) antibody titer ≦1:40 (seroprotection titer) 14 days post-vaccination for H1N1 and H3N2 antigens; number and percentage of subjects seroconverting for H1N1 and H3N2 antigens (seroconversion was defined as a serum HAI titer on day 14 meeting one of the following criteria: (1) pre-vaccination titer <1:10 and post-vaccination titer ≧1:40 or (2) pre-vaccination titer ≧1:10 and at least a 4-fold increase in post-vaccination titer.^[20]^

### 2.5 Hemagglutination and hemagglutination inhibition (HI) test

HAI titers were determined at baseline and day 14.^[21]^ Blood samples (volume, 5 ml) were collected before and 14 days after vaccination. Plasma samples were aliquoted and stored at −80°C before used in the HI assay. Blood samples were obtained and then centrifuge separates the serum from blood, and stored at −80°C until ready to use. Plasma samples from each individual were tested in duplicate by means of an HI assay, using 8 hemagglutination units of the homologous H1N1 and H3N2 vaccine strains and 0.7% turkey red blood cells. HAI titers were defined as the reciprocal of the dilution causing 50% HAI. Negative titers were assigned a value of 5 for calculation purposes. The HI test was completed in the virology laboratory of Wuhan Centers for Disease Prevention and Control.

### 2.6 Screening of cytokines level using flow cytometry

Changes in serum cytokines following vaccination with trivalent influenza vaccine were compared at baseline and day 14.^[22]^ One milliliter blood was collected from study participants into heparin vacutainers (Shanghai Biochemistry Pharmaceuticals Company, Shanghai, China). The blood was diluted 1:4 with RPMI culture (Gibco BRL, CA) containing 2 mm l-glutamine, 100 U/ml penicillin, and 100 μg streptomycin. The diluted blood was subsequently incubated at 37°C in 5% CO_2_ for 2 days without any exogenous stimulation. The culture supernatants were harvested into cryovials and stored at −80°C until use.

The Cytometric Bead Array (CBA) Human Th1/Th2/Th17 Cytokine Kit (BD Biosciences, San Jose, CA) was used to measure IL-2, IL-4, IL-6, IL-10, TNF, IFN-γ, and IL-17 protein levels in a single sample. Seven bead populations with distinct fluorescence intensities have been coated with capture antibodies specific for IL-2, IL-4, IL-6, IL-10, TNF, IFN-γ, and IL-17 proteins. The 7 bead populations are mixed together to form the bead array. Test samples and PE detection antibody were incubated with capture bead reagent for 3 hours in the dark at room temperature. All unbound antibodies are washed (1.0 ml wash buffer), re-suspended in 300 μl before acquisition on BD FACS array bio-analyzer (BD Bioscience, San Jose, CA). All 7 individual cytokine standard curves (range 20–5000 pg/ml) were run in each assay. The microparticles were resuspended in buffer and read using a BD FACS Array bio-analyzer by Union Hospital, Tongji Medical College of Huazhong University of Science & Technology.

### 2.7 Data source

Two researchers reviewed and abstracted the data. All identifiable personal information was removed for privacy protection. Data were entered into a computerized database and cross-checked. If the core data were missing, requests were sent to the clinicians. All date were available at http://www.chictr.org.cn/index.aspx (Registration number: ChiCTR1800019602).

### 2.8 Statistical analyses

All calculations were performed using SAS version 9.1 (SAS Institute Inc., Cary, NC), figures were drawn by Prism 5 software (GraphPad Software, Inc). Safety endpoints were assessed in the safety analysis set. The difference of frequency rates between 2 groups were compared by chi-square test or Fisher Exact test or Cochran–Mantel–Haenszel test. The statistical significance of differences among cytokine levels was determined using the nonparametric Kruskal–Wallis test.

## 3 Results

### 3.1 Participants

**Figure 1:**
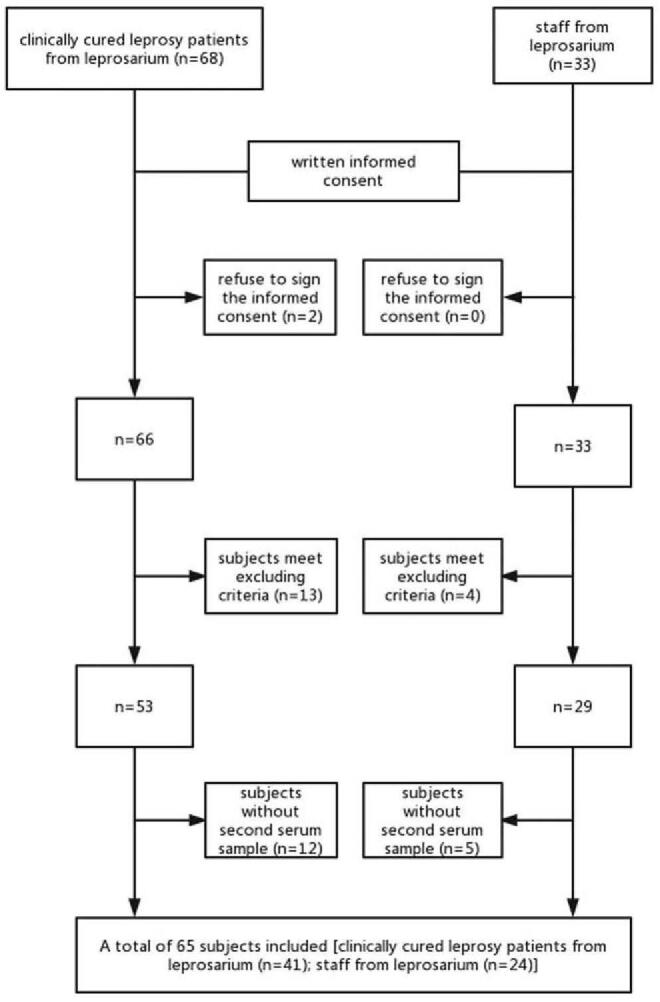
Flow chart.

As shown in Figure 1, between November 21, 2017 and December 1, 2017, a total of 101 subjects were screened for eligibility, 65 subjects were included in the study. All participants enrolled received a 0.5 ml dose vaccination and a 28 days follow-up. Leprosy cured group had a higher proportion of male than control group. The mean age of participants was 72.17 in leprosy cured group and 44.29 in control group. All participants are of race Han (Table 1). All leprosy cured patient were treated at Wuhan leprosarium and the mean time release from treatment is 32 ± 6.17 years.

**Table 1:** Population demographics.

Characteristic	Leprosy cured group (n = 41)	Control group (n = 24)	*P*
Sex, n(%)			
Male	32 (78.05)	8 (33.33)	<.0001
Female	9 (21.95)	16 (66.67)	
Age (yr), mean ± SD	72.17 ± 7.18	44.29 ± 9.80	<.0001
Race, n (%)			
Han	41 (100)	24 (100)	–
Other	0 (0)	0 (0)	

### 3.2 Safety and tolerability

The total rate of reactogenicity was 0.00% [0/41] in leprosy cured group and 37.5% [9/24] in control group (Table 2). In control group, the most common local reaction was itching and the most common solicited systemic reaction was fever. No recurrence of lepra reaction, immediate unsolicited SAEs, and AEs leading to early withdrawal from the study, or deaths were reported in this study.

**Table 2:** Number of subjects experienced adverse reactions within 28 d of vaccination.

	Leprosy cured group (n = 41)		Control group (n = 24)	
Variables	Male	Female	Male	Female
Solicited injection-sites adverse events				
Subtotal^∗^	0	0	3	4
Pain	0	0	1	0
Itching	0	0	2	3
Erythema	0	0	1	0
Swelling	0	0	0	1
Induration	0	0	0	1
Ecchymosis	0	0	0	0
Solicited systemic adverse events				
Subtotal^∗^	0	0	2	5
Fever	0	0	1	3
Malaise	0	0	0	1
Headache	0	0	1	1
Myalgia	0	0	0	0
Vomiting	0	0	0	0
Diarrhea	0	0	0	0
Shivering	0	0	0	0
Total^∗^	0	0	3	6

### 3.3 Immunogenicity and seroconversion rate

Fourteen days after vaccination, the seroconversion rate was 79.17% for H1N1, 50.00% for H3N2 in control group, compared to 41.83% and 34.25% in leprosy cured group (Table 3). The difference of seroconversion rate between the 2 groups was particularly pronounced for the H1N1 strain (*P* = .0082). We divided leprosy patients into 2 subgroups according to the leprosy type. TL and borderline TL were considered as TT, and LL, borderline LL and midborderline leprosy were considered as LT. Statistical analysis detected no significant difference of seroconversion rate in either H1N1 or H3N2 between 2 subgroups.

**Table 3:** Seroconversion rates at 14 d after vaccination.

group	H1 seroconversion rate % (95%CI)	*P*	H3 seroconversion rate % (95%CI)	*P*
Control (N = 24)	79.17 (57.85–92.87)	.0032	50.00 (29.12–70.88)	.2080
Leprosy cured (N = 41)	41.46 (26.32–57.89)		34.15 (20.08–50.59)	
LT (N = 18)	38.89 (17.30–64.25)	.7672	27.78 (9.69–53.48)	.4468
TT (N = 23)	43.48 (23.19–65.51)		39.13 (19.71–61.46)	

### 3.4 Cytokine level

**Figure 2:**
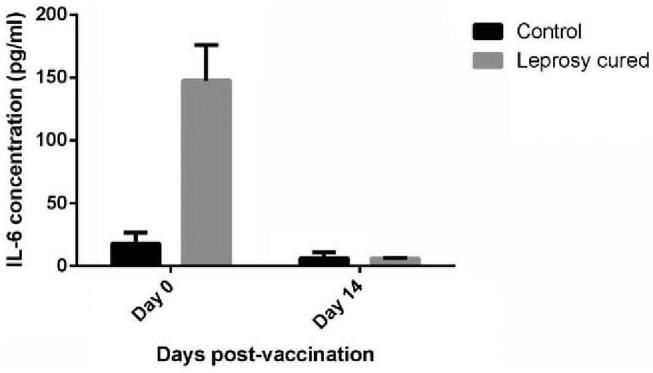
Comparison of pre-2 week IL-6 concentration change between 2 groups.

We further select 7 cytokines and immune mediators to detect immune response after vaccination and found that before the onset of vaccination, leprosy cured group have relatively high concentration of IL-6, IL-10, TNF, INF-γ and IL-17 compared to control group (Table 4). The IL-2 concentration declined 2 weeks after vaccination compared to pre-vaccination in control group, but increased in leprosy cured group (−0.02 pg/ml vs 0.92 pg/ml, *P* = .0147); the IL-6 concentration declined more in leprosy cured group when influenza virus was challenged (−11.52 pg/ml vs −144.38 pg/ml, *P* < .0001) (Table 5; Fig. 2).

**Table 4:** Serum cytokines concentration before vaccination (pg/ml).

Group	IL-2	IL-4	IL-6	IL-10	TNF	INF-γ	IL-17
Control (N = 24)	3.17 ± 6.11	1.15 ± 1.59	17.98 ± 42.21	1.21 ± 1.02	1.63 ± 5.14	0.1 ± 0.19	2.74 ± 6.69
Leprosy cured (N = 41)	2.32 ± 1.13	1.42 ± 1.07	147.41 ± 183.11	2.73 ± 2.08	3.22 ± 2.56	1.67 ± 2.21	10.45 ± 14.76
*P*	.1477	.1041	<.0001	<.0001	<.0001	.0003	.0019
LT (N = 18)	1.99 ± 1.08	1.32 ± 1.18	119.64 ± 126.44	2.85 ± 2.67	2.49 ± 2.21	0.97 ± 1.22	5.4 ± 7.33
TT (N = 23)	2.57 ± 1.14	1.49 ± 0.99	169.14 ± 217.9	2.63 ± 1.53	3.79 ± 2.72	2.22 ± 2.64	14.4 ± 17.82
*P*	.1243	.5194	.5812	.5721	.0852	.0393	.1280
Ulcer (N = 29)	2.29 ± 1.16	1.52 ± 1.28	131.44 ± 144.49	3.39 ± 3.06	2.56 ± 1.94	1.59 ± 2.78	7.83 ± 16.09
Non-ulcer (N = 12)	2.33 ± 1.14	1.38 ± 0.99	154.01 ± 198.85	2.45 ± 1.5	3.49 ± 2.76	1.7 ± 1.98	11.53 ± 14.34
*P*	.5763	.9886	.7745	.2457	.439	.5372	.1757

**Table 5:** Serum cytokines concentration change from day 0 to day 14 after vaccination (pg/ml).

Group	IL-2	IL-4	IL-6	IL-10	TNF	INF-γ	IL-17
Control (N = 24)	−0.02 ± 3.82	−0.26 ± 1.27	−11.52 ± 21.16	0.42 ± 2.41	0.05 ± 4.57	0.01 ± 0.3	0.11 ± 5.71
Leprosy cured (N = 41)	0.92 ± 1.58	0.58 ± 1.51	−141.38 ± 184.02	−0.15 ± 1.99	−1.12 ± 2.28	−0.47 ± 1.43	−1.81 ± 11.8
*P*	.0147	.0784	<.0001	.5773	.09	.1898	.191
LT (N = 18)	1.13 ± 1.7	0.65 ± 1.64	−113.85 ± 127.12	−0.27 ± 2.48	−0.5 ± 1.94	0 ± 0.99	2.89 ± 7.1
TT (N = 23)	0.76 ± 1.49	0.52 ± 1.44	−162.92 ± 219.04	−0.07 ± 1.55	−1.61 ± 2.46	−0.84 ± 1.63	−5.48 ± 13.49
*P*	.2994	.7928	.5993	.8644	.1761	.091	.0065
Ulcer (N = 29)	0.97 ± 1.91	0.44 ± 1.7	−124.04 ± 146.25	−1.04 ± 2.8	−1.23 ± 1.65	0 ± 1.48	0.5 ± 8.73
Non-ulcer (N = 12)	0.91 ± 1.46	0.63 ± 1.46	−148.55 ± 199.48	0.21 ± 1.45	−1.07 ± 2.52	−0.67 ± 1.39	−2.76 ± 12.87
*P*	.9315	.9088	.7745	.1024	.4916	.2997	.4823

**Figure 3:**
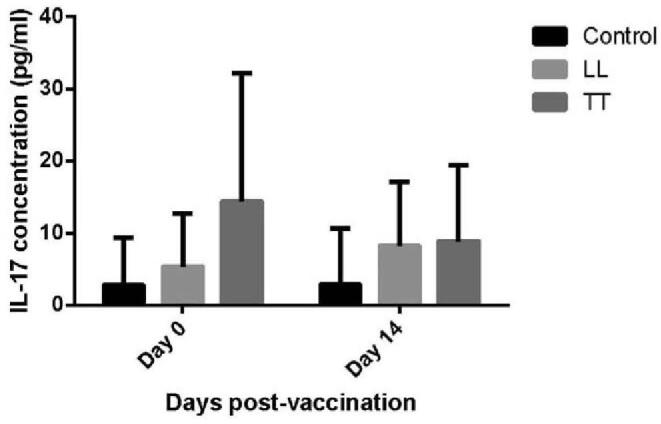
Comparison of pre-2 week IL-17 concentration change between 3 groups.

Subgroup analysis divided leprosy cured patient into 2 subgroups by leprosy type or whether suffering ulcer. Result revealed that the immunization administration declined IL-17 concentration in TT type subgroup, but not in LL type subgroup or control group (*P* = .0065) (Fig. 3). IL-17 was down-regulated in TT type subgroup group while vaccination onset (from 14.40 pg/ml before vaccination to 9.92 pg/ml after vaccination), but up-regulated in LL type subgroup group (from 2.74 pg/ml before vaccination to 2.85 pg/ml after vaccination) and control group (from 5.40 pg/ml before vaccination to 8.29 pg/ml after vaccination).

## 4 Discussion

Our previous study conducted an observational clinical study to evaluate the safety of influenza vaccine in elderly clinically cured leprosy patients.^[23]^ The acceptability of influenza vaccination increased from 58.4% during the same period last year to 97.1% in this study. Being treated equally in medical care, persons affected by leprosy may reduce their self-stigma and discrimination.^[23,24]^ The rate of reactogenicity was low in leprosy cured group, correspond to our previous study, that influenza vaccine had a relatively good tolerability in clinically cured leprosy patients. In this study, we conduct a more proactive safety monitoring strategy with medical professional examine instead of self-report, to deal with perceive less uncomfortable sense of injection site of leprosy cured person due to tissue damage and suffer sensory loss caused by *Mycobacterium*.^[25,26]^ Based on the evidence of safety monitoring, we concluded that leprosy affected persons are relatively safe for influenza vaccinations.

The immunology of vaccines in leprosy affect person is unclear due to poor evidence. In the present study, we performed a HI assay to evaluate protective effect of influenza vaccine. Result suggested that leprosy cured patient have relatively weaker H1N1 response, implied that cured leprosy patients have immune deficit in producing antibody, compared to normal people. The result of H3N2 response suggested that the difference between 2 groups is not significant. This result can be explained by relatively small sample size, or the mutation of influenza vaccine. H3N2 remained stable but H1N1 showed variation compared with last year. The vaccine was provided in compliance with World Health Organization recommendations, modified A/California/7/2009(H1N1) pdm09-like virus to A/Michigan/45/2015 (H1N1)pdm09-like virus, but not H3N2 and B strain.^[17]^ Consequently, H3N2 response in the present vaccine immunization may not be a new response to antigenic stimulation, and may not completely reflect immune status. In any case, cured leprosy patients expressed lower immune response within 2 weeks after the onset of vaccination. This may related to the immune deficiency or immunologic derangement of cured leprosy patients. Genetic variations of genes in the NOD2-mediated signaling pathway are associated with susceptibility to infection with *M. leprae* had been identified in leprosy patients.^[27,28]^ NOD2 and RIPK2 interact to activate the NF-κB pathway, which affects the host's immune defense against infection.^[29,30]^ Another possibility is that *M. leprae* damaged host immune system, reconstructed host immune microbial ecology to a new balance, even if *M. leprae* were removed,^[12,31,32]^ which lead to the lower immune response to vaccination. Result of cytokine analysis may support this hypothesis. Cytokines level before the introduction of vaccination suggested that leprosy cured group have relatively high concentration of IL-6, IL-10, TNF, INF-γ and IL-17 compare to control group. These results correspond to the previous study that leprosy, or leprosy cured patient are immunologic derangement.^[31,33–37]^ Dysregulated continual synthesis of these cytokines can be considered as consequent of *M. leprae* infection. Hyperinflammation promote the elimination of pathogens in early period of invasion, lead to lower antibody response towards inactivated influenza virus. As pro-inflammation such as IL-6 and IL-17 were largely consumption while host defense, the declining concentration of pro-inflammation cytokines were observed. The lower level of inflammatory responding to immune triggers in leprosy group may partly explain lower risk of adverse outcomes above. To further improve the immunogenicity of influenza vaccine in leprosy cured patients, intradermal vaccine, high dose vaccine, nasal spray live attenuated vaccine may be considerable.^[38]^

It is generally accepted that leprosy is an infectious disease associated with the immune function of the organism.^[30,39]^ In the process of leprosy immunity, many physiological effects of cytokines are mutually influenced and restricted, together form a complex network.^[39]^ It is important to maintain the network balance, in other words, the body's immune stability. Any intervention factors break this balance are likely to lead to the body's immune disorder, thereby cause the development of diseases.^[31,40]^ IL-6 is a pro-inflammatory cytokine secreted by T cells and associated with the development of erythema nodosum leprosum (ENL), participated in the process of immune regulation by activated CD4+ T cells and in the final differentiation of B-cells into Ig-secreting cells.^[41,42]^ Although humoral immune responses have been traditionally associated with protection against influenza, CD4+ T cells also play crucial roles in immunity to influenza, their contribution to protection against influenza has been reported in humans and animal models.^[43–45]^ Recently, researchers have shown more and more interest in the function played by cytokine in inflammatory complication damage caused by viral diseases. The latest retrospective study reviewed 150 confirmed COVID-19 cases, found that sharp increases in ferritin and IL-6 in non-survivors, suggesting that the deaths may have been caused by virally driven hyperinflammation.^[46]^ The rapid production of IL-6 mediate lymphocyte infiltration and activation in the pneumonic lung, which might leads to acute respiratory distress syndrome.^[47,48]^ It is interesting that under the influence of host's immune response elicited by vaccination, the degree of decrease of IL-6 was 14-fold in leprosy cured group than in control group. The significant down-regulated of IL-6 concentration in leprosy cured patients suggested that influenza vaccination elicited inhibitory mechanism of inflammatory, break the balance of host's existing immune network. Down-regulated of IL-6 let host vulnerable to infection, subsequently raise concern on recurrence of leprosy clinical manifestations and other diseases.^[49–51]^ But during 28 days safety monitoring, the onset of vaccination did not result in leprosy reactions or any other safety event. The sharp fluctuation of IL-6 concentration in leprosy affected person different from normal people has potential for leprosy treatment, and can better predict disease in leprosy early monitoring, as it has been used in acute myocardial infarction and other disease.^[52]^

The difference of IL-17 concentration between 3 groups suggested the immune difference between Lepromatous and Tuberculoid leprosy type. The TT and LT divergence can be characterized by host immunity. The CD4+ T cell predominates in TT, leading to a Th1 cytokine profile, while there is a greater antibody response in patients with LT. In recent times, the IL-17 cytokines are emerging as key player in immune responses.^[53]^ IL-17 is uniquely secrete by Th17 cells, which are critical to the adaptive immune response against bacterial and fungal infections, and also contribute to the pathogenesis of several inflammatory diseases, by recruiting neutrophils, activating macrophages, and enhancing Th1 effector cells.^[54]^ IL-17 has also been observed as associated cytokine with leprosy reactions.^[55]^ TL patients with type 1 reactions (T1R) showed down-regulate of IL-17. On the other hand, LL with ENL showed an increase in IL-17.^[33,56]^ IL-17 also plays a role in the early infection and prevention of leprosy, as study find out that healthy household contacts with a long-term exposure to patients have a higher expression of IL-17 compared with unexposed individuals.^[57]^ IL-17 as a sensitive biomarker for TL type may helpful for leprosy monitoring and treatment.

Our study has several limitations need to be addressed. First, the disparity between the leprosy and control groups in respect of age may lead to potential bias, as this could be potential reason for differences in immune response and antibody levels. Second, cured leprosy patient recruited has been cured for more than 20 years, it is hard to identify antibodies to *M. leprae* antigen. Third, the antibodies to *M. leprae* antigen have not compared, it is difficult to clarify if defects are general or specific. Finally, cytokine bioactivity was obtained for pre-vaccination to 2 weeks after vaccination, a long term observation of cytokine level change is needed to better investigate the role of cytokines.

To our knowledge, the present study is the first clinical study to evaluate the immunogenicity and safety of influenza vaccine in clinically cured leprosy patients. We concluded that clinically cured leprosy patients are relatively safe after being immunized with influenza vaccine, and persons affected by leprosy should be treated equally in medical care, thus relieve their psychological burden and promote their social engagement. We found that leprosy cured patient have immune deficit in producing antibody, compared to normal people. We also identify IL-6 and IL-17 as 2 sensitive indicators of immune response in leprosy cured patients. The immunogenicity assessment of the study could enhance our comprehension of complex network created by cytokine release, and provide data that will help in the development of new strategies for leprosy management.

## Author contributions

Yi Zheng analysis of data and drafted the work; Hong-yi Xing has finished laboratory test and drafted the work. Zheng-Gang Zhu interpretation of data and have drafted the work; Hong-Hao Zhu and Fang Zhang have finished laboratory test; Xia Gao and Jun Gao acquisition of data; Quan Hu and Yuan Fang design of the work and revised the manuscript.

**Data curation:** Yi Zheng, Hong-yi Xing, Fang Zhang, Xia Gao, Jun Gao.

**Formal analysis:** Hong-Hao Zhu, Hong-yi Xing, Fang Zhang.

**Funding acquisition:** Quan Hu.

**Investigation:** Xia Gao, Jun Gao.

**Methodology:** Hong-Hao Zhu, Quan Hu, Yuan Fang.

**Project administration:** Yuan Fang.

**Software:** Yi Zheng.

**Writing – original draft:** Yi Zheng, Hong-yi Xing, Zheng-Gang Zhu, Quan Hu, Yuan Fang.

**Writing – review & editing:** Yi Zheng, Hong-yi Xing, Zheng-Gang Zhu, Quan Hu, Yuan Fang.
